# Dynamics of microbial populations and metabolites of fermenting saps throughout tapping process of ron and oil palm trees in Côte d’Ivoire

**DOI:** 10.3389/fmicb.2022.954917

**Published:** 2022-10-31

**Authors:** Theodore N. Djeni, Santosh Keisam, Karen H. Kouame, Christelle Nanouman Assohoun-Djeni, Francine D. M. Ake, Laurent S. T. Amoikon, Ngangyola Tuikhar, Rajendra K. Labala, Marcellin K. Dje, Kumaraswamy Jeyaram

**Affiliations:** ^1^Laboratoire de Biotechnologie et Microbiologie des Aliments, Unité de Formation et de Recherche en Sciences et Technologie des Aliments (UFR-STA), Université Nangui Abrogoua, Abidjan, Côte d'Ivoire; ^2^Microbial Resources Division, Institute of Bioresources and Sustainable Development (IBSD), Takyelpat Institutional Area, Imphal, Manipur, India; ^3^Unité de Formation et de Recherche en Agroforesterie, Université Jean Lorougnon Guédé de Daloa, Daloa, Côte d'Ivoire

**Keywords:** *Borassus aethiopum*, dynamics, *Elaeis guineensis*, gevotroline, health benefit, microbiota, palm wine tapping

## Abstract

Palm wine fermentation is a complex microbial process that evolves with tapping times. The dynamics in microbiota and metabolites throughout palm wine tapping days is still not established, which are critical for the distinctive characteristics of palm wine taste and quality, and thus the mastery of the daily quality fluctuation during tapping. We analyzed the changes in microbial community structure by amplicon sequencing of bacterial 16S rRNA gene and fungal internal transcribed spacer (ITS) region, and metabolite profile changes using mass spectrometry in palm wine collected over 25–30 days tapping of ron (*Borassus aethiopum*) and oil palms (*Elaeis guineensis*) from Côte d’Ivoire. The stage-wise collected palm wine samples showed distinct changes in microbial diversity and pH, supporting microbial community dynamics during palm wine tapping. Results highlighted the dominance of *Saccharomyces cerevisiae* in early stages and the emergence of non-*Saccharomyces* yeasts, particularly *Hanseniaspora* spp. in the later stages of oil palm wine tapping, vice versa in the case of ron palm wine tapping, with a unique presence of *Saccharomycodes* in the later stages (15–30 days). Fructophilic lactic acid bacteria (FLAB), mainly *Fructobacillus* and *Leuconostoc*, encountered in both types of palm wine tapping showed a decline at later stages of oil palm wine tapping. In this type of palm wine, acetic acid bacteria with genera *Acetobacter* and *Glucanoacetobacter*, by surpassing *Lactobacillus* in the last stage become dominant, whereas *Lactobacillus* remained dominant in ron palm wine throughout tapping days. The decline in the relative abundance of gevotroline and essential amino acids during the later stages of palm wine tapping (15–25 days) supports the difference in the health benefits of the palm wine obtained from different days of tapping, indicating that early stages of tapping is more nutritional and healthy than the later stages. The microbial dynamics may be a potential indicator of metabolite changes during palm sap fermentation, thus contributing to establish particular features of palm wines in different stages of tapping. This understanding of microbial ecology and chemical composition changes during palm wine tapping can be used as biomarkers to assess palm wine’s quality and help to design an optimum starter culture.

## Introduction

West African forests cover an area of ~75 million hectares (FAO, 2003), and are characterized by exceptional biological diversity. This plant diversity is made up of a large proportion of non-timber forest products (NTFPs), which are defined as goods of biological origin other than timber and services derived from forest and related land use systems (FAO, 1995). The exploitation and sale of these products provide significant income, especially for the most vulnerable rural and urban populations. For example, in Central Africa, NTFPs play a multiple role for about 65 million people living in or near forests, notably as sources of energy, food, medicines and service products. In Côte d’Ivoire, among these plants, palm trees, notably oil palm (*Elaeis guineensis*), ron (*Borassus aethiopum*) and raffia (*Raphia* sp.) occupy an important place by providing in addition to several food products (seed, mushrooms, saw palmetto, etc.), a very popular drink named “palm wine.” This drink constitutes with the red oil of the fruit of oil palm in central and southern regions of Côte d’Ivoire, the most important products of gathering (Mollet et al., 2000).

**Figure 1:**
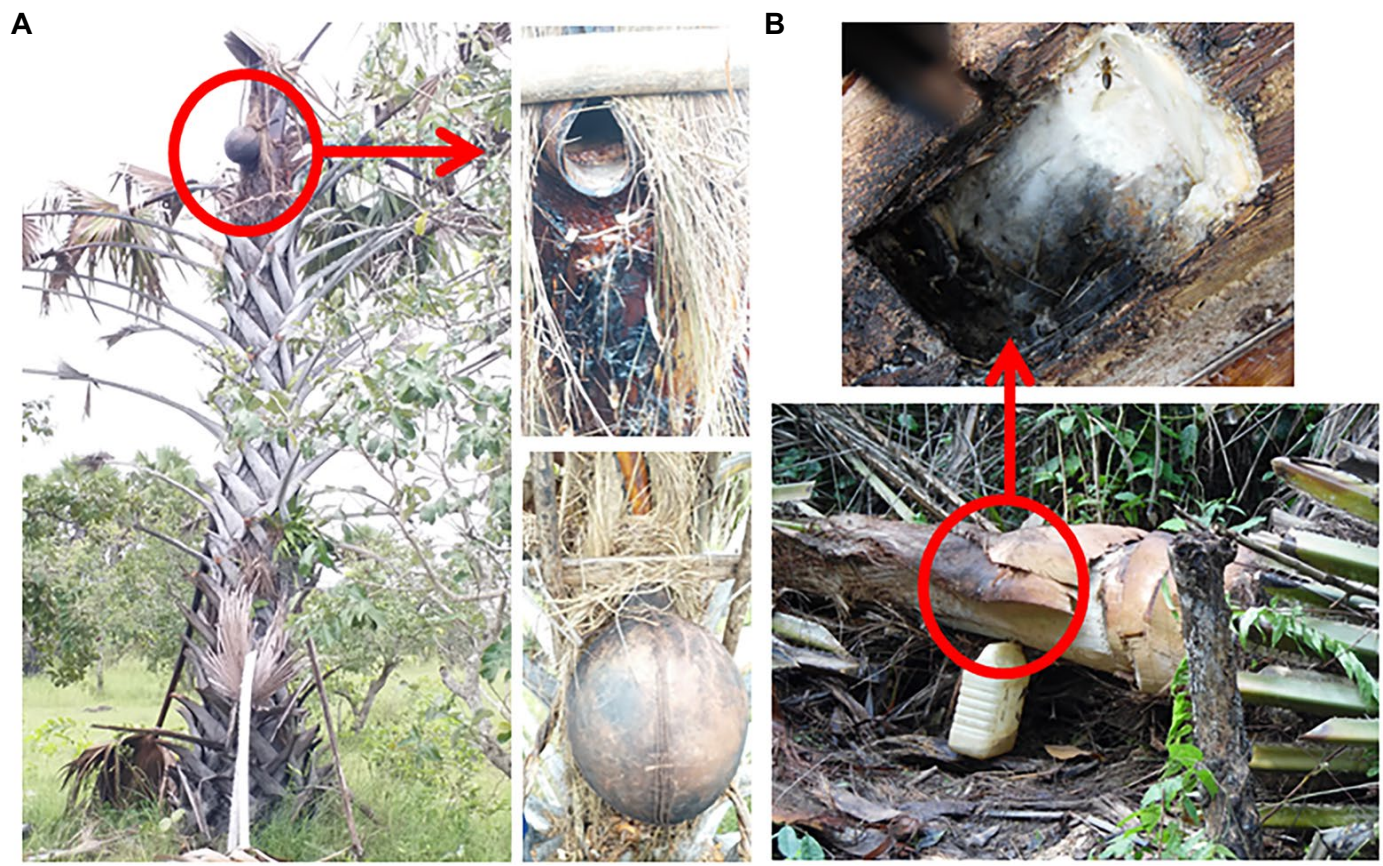
The indigenous processes of palm sap tapping practiced in Côte d’Ivoire for palm wine production from ron palm (*Borassus aethiopum*) **(A)** and oil palm (*Elaeis guineensis*) **(B)**. Perforation of the apical meristem of the tree trunk is in practice for sap tapping from ron palm, whereas the oil palm tree is uprooted before tapping.

“Palm wine” is a natural drink sweetened at harvest and alcoholic after spontaneous fermentation. This traditional alcoholic drink, widely consumed in several African countries, remains among the most popular drinks and are culturally rooted in the practices of people in Africa for traditional rituals. Generally consumed outside meals, it plays an important role in the nutrition of rural populations (Lucky et al., 2017; Erukainure et al., 2019). Indeed, far from being the only fact of pleasure, the consumption of “palm wine” is an important element of rural population’s life during lean periods as well as during intense field works. Therefore, this drink plays a vital role in the traditional food culture of societies living in these regions, with regards to its involvement in traditional ceremonies, notably traditional namings and marriage ceremonies, traditional incantations in African communities (Lucky et al., 2017; Djeni et al., 2020). Several traditional techniques for extracting palm wine in Africa exist. These practices known as “tapping” have been widely described by many researchers (Blanc-Pamard, 1980; Mollet et al., 2000; Kadere et al., 2004; Onuche et al., 2012; Ouoba et al., 2012). However, two techniques (on a living palm tree and on on felled palm trees) are mainly used in Côte d’Ivoire (Figure 1). Once tapping process is set up, the first drops of sap automatically undergo spontaneous fermentation, with the conversion of the sweet substrate into an alcoholic beverage containing important nutritional components.

The spontaneous fermentation of the palm sap leads to the growth of various indigenous microorganisms and the release of a multitude of metabolites. Indeed, the interactions of microbial consortia in fermented foods promote the process of polymer degradation and the production of compounds of interest which contribute to functional and organoleptic properties (Das and Tamang, 2021). It is recognized that metabolites such as organic acids (lactic and acetic acids) and other flavoring compounds (esters and amino acids) are important components in palm wine tastes and flavors and metabolite profiles represent a collective phenotypic view that reflects palm wines microbial communities and consequently the overall quality of these beverages. Due to various considerations, notably nutritional, medicinal importance, inexpensiveness, health promoting benefits and easy availability, many research programs have been developed on palm wine, with respect to multiple aspects such as stability, physiological/nutritional changes, biochemical constituents, microbial flora associated physiochemical changes, metal profile, pasteurization and additives, and role of preservatives in shelf life (Doddipalla et al., 2021). However, there is a dearth of information in understanding microbial and metabolic changes during the whole tapping process of various palm trees and research attempts should be planned to establish the daily quality of palm wine throughout tapping duration, with regard of beneficial aspects in one hand and in other hand to consumer health insurance. The few studies carried out with respect to tapping times presented a superficial view of quality variations, due to the fact that the analytical techniques used were not state-of-the-art and that only few physico-chemical parameters (pH, titratable acidity, certain organic acids) and microbiological (count of lactic acid bacteria, acetic acid bacteria and yeasts) were considered (Amoa-Awua et al., 2007; Karamoko et al., 2012). This still leaves a lot of gray areas as to the understanding of the benefits that this drink provides to consumers and especially the tapping period that provides the most benefits.

Moreover, in the recent times, palm wine market begins to be negatively affected due to the occurrence of bad practices in some production areas. In fact, to maximize their profit margin, some tappers and/or vendors are engaged in various forms of palm wine manipulations, which lead to adulteration of the product. Thus “palm wine” is diluted with unfiltered water by vendors and to maintain the required taste, substances such as sugars are added. Some of the consequential product of adulteration is presumed to contain salicylate and high alcoholic content as reported by Bisi-Johnson et al. (2011). Such compounds are known to have toxic effect at a certain level of exposure with a ripple health effects. Based on the high consumption of palm wine in Côte d’Ivoire, coupled with the fact that some unscrupulous retailers use various chemicals of unknown composition for artificial production of palm wine, and as little is known about the metabolome and the microbiota of the palm wine types, omics techniques appear to be necessary to provide insight for documenting the unexplored biodiversity and ecological characteristics of either whole communities or individual microbial taxa, with the high possibility of finding technologically promising molecules or species and/or predicting the safety of this beverage.

The detection of palm wine metabolites were primarily achieved using traditional methods such as high-performance liquid chromatography and gas chromatography, but it seems almost difficult to simultaneously analyze all palm wine metabolites using these methods, especially during the whole period of tapping, because a wide variety of metabolites, having different chemical properties, are produced during the sap fermentation. Nowadays, metagenomics and metabolomics are gaining importance in the majority of the research platforms, especially in biomarker detection, disease diagnosis, cellular metabolism, food quality and global profiling approaches to recognize the intrinsic variability in microbial communities and metabolic path ways by applying high throughput sequencing, spectrometry and spectroscopic techniques (Zhang et al., 2013; Li et al., 2021).

Since microorganisms are essential for the distinctive biochemical characteristics of palm wines, the growing popularity and demand for this drink, particularly in urban areas requires a better understanding of the roles of these microorganisms in palm wine production, its quality and the basis of its beneficial effect on health. Therefore, in this study, we applied a combination of a next-generation sequencing technique (Illumina miseq) and metabolite analyses using ultrahigh performance Liquid chromatography (UHPLC)/mass spectrometry to examine the changes in the microbiota and metabolites of naturally fermented saps collected during palm wine tapping over a period of 25–30 days from ron palm (*Borassus aethiopum*) and oil palm (*Elaeis guineensis*); and then determine whether during the whole tapping process, the beverage appears to be more nutritional and beneficial to consumers health.

## Materials and methods

### Sample collection

The samples of palm wine were obtained from two palm tree species, namely *Borassus aethiopum* (ron palm) and *Elaeis guineensis* (oil palm) during the small rainy season (october to november) in 2016. The fermented saps from *Borassus aethiopum* were collected on five (5) trees at Toumodi in the center of the country (6°33′28″N, 5°01′03″W), while those of *Elaeis guineensis* were collected on three (3) trees at Botiji, a village of Adzope town (6°6′25″N, 3°51′42″W) in Côte d’Ivoire. Samples were collected one time per day by transferring the content of producer’s collection pot after shaking it directly into a sterile container that we provide to him. And this occured early in the morning (7:00 a.m.) at 10 different stages of tapping (1st, 2nd, 3rd, 5th, 7th, 10th, 15th, 20th, 25th and 30th day). In total, 45 samples of ron wine and 30 samples of oil palm wine were collected in sterile containers, stored immediately in a cooler containing cold packs and transported to the laboratory for physicochemical and microbiological analysis within up to 2 h. Once at the laboratory, 30 ml of each sample were centrifuged at 12,000*g*. The pellets were washed twice with sterile saline water, resuspended in the same liquid with 30% glycerol and kept at −20°C until microbial community analyses. The supernatants were also stored at −20°C for metabolomics analyses.

### Metagenomic DNA extraction

An aliquot of 0.25 ml of each resuspended pellet was homogenated with 1 ml of sterile water in a sterile 2 ml screw-cap tube containing 0.5 g of zirconia/silica beads (0.1 mm) and 4 glass beads (2 mm) and total microbial DNA was extracted using beads beating technique combined with phenol-chloroform purification (Djeni et al., 2020). The quality and quantity of the extracted DNA were firstly assessed by 0.8% agarose gel electrophoresis, and then by the OD 260/280 measurement using a NanoDrop spectrophotometer ND-1000 (Thermo Scientific, USA). All of the DNA samples were stored at −20°C until further processing.

### Library construction and Illumina MiSeq sequencing

The V3-V4 region of the 16S ribosomal RNA gene for bacteria and the ITS2 region of fungi were PCR amplified from each sample. A set of 8-bp barcodes for bacteria and 12 bp for fungi were added to the bacteria universal forward primer 5′-AYTGGGYDTAAAGNG-3′ and reverse primer 5′-CCGTCAATTCMTTTRAGT-3′ (Romi et al., 2015) and fungi forward primer ITS1-F: 5′-CTTGGTCATTTAGAGGAAGTAA-3′ and reverse primer ITS2: 5′-GCTGCGTTCTTCATCGATGC-3′ (White et al., 1990; Motooka et al., 2017) respectively. PCR reactions and conditions were similar to those applied by Djeni et al. (2020). The PCR amplicons were then separated in a 2.0% agarose gel (w/v), purified using QIAquick gel extraction kit (Qiagen, New Delhi, India) and quantified using Qubit dsDNA BR Assay Kit (Invitrogen) in a Qubit 2.0 fluorometer (Invitrogen, Carlsbad, CA). The individual amplified samples were pooled together in equimolar ratios. These obtained pools were sequenced with paired-end Illumina MiSeq by the NGS facility “Xcelris Genomics” (Ahmedabad, India).

### Processing of sequencing data

The raw nucleotide sequence reads obtained from MiSeq amplicon sequencing were processed using the QIIME v1.8.0 bioinformatics pipeline (Caporaso et al., 2012). The adaptor sequence removal, generation of paired-end reads, and sample demultiplexing were carried out as mentioned in Romi et al. (2015). The segregation of yeast and bacterial sequences was performed using in-house Perl scripts. The segregated were separately clustered and taxonomically identified as operational taxonomic units (OTUs) at 97% identity threshold by using the furthest-neighbor algorithm. For yeast identification, the representative OTU sequences were taxonomically annotated using the UNITE fungal ITS database release version 7.1 (Nilsson et al., 2019; http://unite.ut.ee). The MG-RAST pipeline was used for taxonomic annotation at 97% similarity threshold against the M5RNA database for bacteria.

### Metabolites analysis using HILIC UHPLC-MS

Metabolites of palm wine samples were analyzed using the Dionex Ultimate 3000 ultrahigh performance Liquid chromatography (UHPLC), coupled with a Q-ExactiveOrbitrap (Thermo Fisher Scientific) from C-CAMP MS Facility, Bengaluru, by following the method of Padma et al. (2016). Samples were prepared by diluting 1 ml of palm wine supernatant in 1 ml acetonitrile (1,1 dilution). The mixture was centrifuged at 18,000*g* for 5 min at 4°C, then filtered through 0.2 μm PTFE filters into a 2 ml septum vial and stored at −80°C for LC–MS analysis. Before analysis, all the samples were thawed in ice and vortexed well. Taurocholate was used as an internal standard for quality control. The chromatography was performed on a hydrophilic interaction liquid chromatography column (HILIC, 5 μ, 150 mm × 4.6 mm, Phenomenex Luna) with a flow rate of 0.4 ml/min and maintained at 40°C. The mobile phase-A contained 5 mM ammonium acetate in water and the phase-B contained 5 mM ammonium acetate in water with acetonitrile in a ratio of 1:9. The run gradient was 100% B to 0% B over a time period of 45 min and regained to 100% B at 46 min, and maintained at 100% B up to 55 min. The Q-Exactive Orbitrap instrument was set up for data acquisition in the full scan/data-dependent scan (FS/DDS) mode in a mass range of 70–750 m/z, alternating between MS and MS/MS scans. The full scan was set from 70,000 to 140,000 resolutions. The run was performed in the negative ionization mode with a spray voltage of 2,500 V, 320°C vaporizer temperature, sheath gas flow rate of 40 arbitrary units, and auxiliary gas flow rate of 10 arbitrary units. The spiked taurocholate was used as an internal standard and later used for intensity normalization of the data. The raw data files were imported into SIEVE 2.2 for the generation of peak list and component extraction, and the Human Metabolome DataBase/Kyoto Encyclopedia of Genes and Genomes (HMDB/KEGG) were used for possible identification of the compounds. From the pooled quality control samples data, the coefficient of variation (CV) was calculated and the data with CV_QC > 20% were removed (Want et al., 2010).

### Statistical analysis

The species-level OTU data of yeast and bacterial sequences were combined for analyzing the overall microbial community structure difference between the palm wine samples collected from three different types of palm tree species. Principal coordinate (PCoA) analysis was performed with the Bray–Curtis dissimilarity and the significance of the difference between the palm wine types based on the relative abundance of species-level OTUs was tested by PERMANOVA test with 10,000 permutations using Bray-Curtis distances in PAST v3.22. The relative abundance data of the bacterial and yeast OTUs at different taxonomic levels (family, genus and species level) were used for performing statistical analysis. The ANOVA, Mann–Whitney test with Bonferroni corrected value of *p* was generated to show the significant changes in the relative abundance of microbial taxa and metabolites during different stages of tapping days. The diversity indices (Chao species richness and Shannon diversity) were calculated using the species level of yeast and bacterial data together in PAST. The LC-HRMS metabolite profile data were log-transformed (log xi + 1) before PCoA analysis, and the metabolites that were significantly different (log2 fold change and *p* < 0.01) over different stages of tapping were listed (Tuikhar et al., 2019).

## Results

### Dynamics in microbial community structure and diversity during palm wine tapping

**Figure 2:**
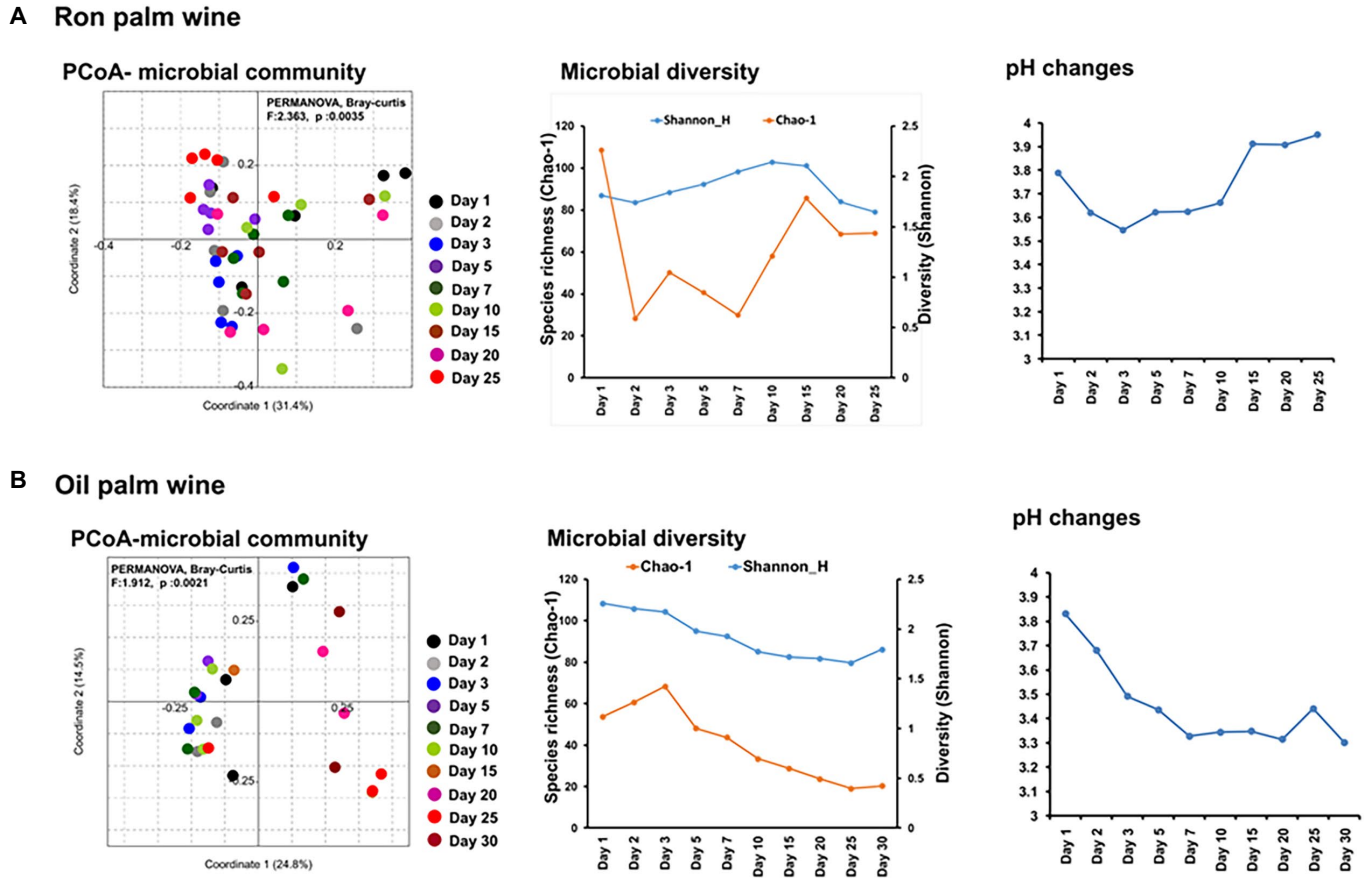
Dynamics in the microbial community structure, diversity and pH during spontaneous fermentation of palm wine tapping from ron palm **(A)** and oil palm **(B)** wine tapping days. PCoA based on Bray–Curtis dissimilarity based on the relative abundance of species-level OTUs, alpha diversity analysis (Chao-1 and Shannon indices) and pH readings of the stage-wise collected samples shows dynamics during 25 days of spontaneous ron palm wine tapping and 30 days of oil palm wine tapping. The significance in the stage wise difference in PCoA is expressed as Bonferroni corrected *p*-values. PERMANOVA: Permutational multivariate analysis of variance.

The bacterial V3-V4 region of 16S rRNA gene and fungal internal transcribed spacer (ITS) region were targeted for MiSeq amplicon sequencing to determine the microbial dynamics during palm wine tapping from ron palm (*Borassus aethiopum*) and oil palm (*Elaeis guineensis*) in Côte d’Ivoire. We analyzed the changes in overall microbial community structure (bacteria and fungi), over 25 days in ron palm wine and 30 days in oil palm wine by sampling at nine stages (*n* = 45 for ron palm wine and *n* = 30 for oil palm wine). This amplicon sequencing resulted in a total of 1,880,438 quality-filtered sequences of 450 bp read length with an average read of 27,653 ± 18,388 per sample for bacteria and 1,225,213 quality-filtered sequences of 200–450 bp read length with an average read of 17,0116 ± 2,063 per sample for yeasts. The taxonomic identification of amplicon sequencing resulted in 193 bacterial genera assigned to 504 species-level OTUs and 95 yeast species-level OTUs involved in the palm wine fermentation. PCoA analysis using Bray–Curtis dissimilarity based on the relative abundance of microbial species-level OTUs profiles showed significant changes during tapping in ron palm wine (*p* = 0.0035, *F* = 2.363, PERMANOVA) and also for oil palm wine (*p* = 0.0021, *F* = 1.912, PERMANOVA; Figure 2). Though a distinct shift in the microbial community structure at different stages was not visible in the PCoA analysis, we could observe an overall change during palm wine tapping. Notably, during the mid-stages of tapping days (7–15 days) in ron palm wine and later stages (15–25 days) in oil palm wine (*p* < 0.05, Pair-wise ANOVA, Bonferroni corrected). Our analysis showed different trends on changes in microbial species richness (Chao-I) and diversity (Shannon index) during ron palm wine and oil palm wine tapping days (Figure 2). The Cho-I index showed a gradual decline over tapping days in oil palm wine, whereas in ron palm wine it noticed a gradual increase up to 15 days, then a drop of diversity index. The ron palm wine had richer microbial diversity than oil palm wine during tapping (*p* < 0.05, ANOVA, Mann–Whitney, Bonferroni corrected). In addition, the pH of ron palm wine increased during the later stages of tapping (pH 3.9, 25 days) while decreased in the later stages of tapping (pH 3.3, 30 days) in oil palm wine fermentation. The changes in pH are significant and positively correlated with microbial species richness, which means low species richness with low pH, during palm wine tapping in both ron palm wine and oil palm wine fermentation (Pearson correlation, *p* < 0.01). The distinct changes in bacterial diversity observed over 25–30 days support microbial community structure dynamics during palm wine tapping days.

### Dynamics in bacterial communities during palm wine tapping

**Figure 3:**
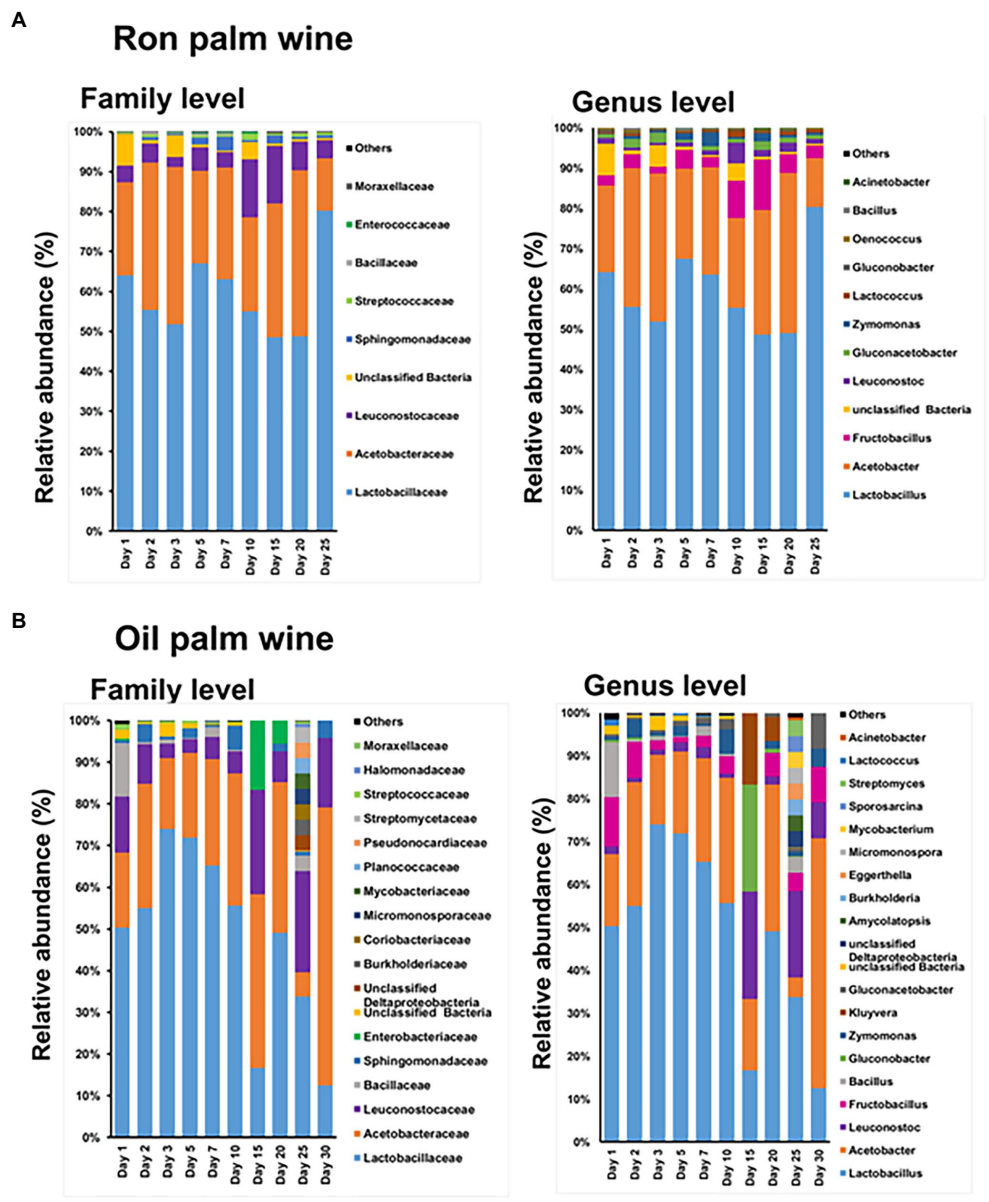
The bacterial taxon bar chart shows the dynamics in the relative abundance (%) of predominant bacteria at family-level and genus level during ron palm wine **(A)** and oil palm wine **(B)** tapping days. The bacterial taxa with a mean relative abundance of <1% across the samples are combined and shown as others.

We assessed the changes in the bacteria composition at different taxonomic levels (family, genus, and species) during the palm wine tapping period by comparing the changes in their relative abundances. The taxonomic analysis showed that *Lactobacilliaceae* and *Leuconostocaceae* of phylum *Firmicutes* and *Acetobacteraceae* of phylum *Proteobacteria* were predominantly present at the family level throughout palm wine fermentation (with a cumulative relative abundance of more than 95% in ron wine and 88% in oil palm wine). At the genus level, *Lactobacillus* of *Lactobacilliaceae*, *Leuconostoc* and *Fructobacillus* of *Leuconostocaceae*, and *Acetobacter* and *Glucanoacetobacter* of *Acetobacteraceae* were present predominantly throughout the tapping days in both palm wine fermentation (Figure 3). In ron palm wine, a significant increase in the relative abundance of *Leuconostocaceae* from 4.4% to 10.7% during 10–15 days of tapping, reflected with a substantial increase in the relative abundance of *Fructobacillus* (Figure 3A), particularly *Fructobacillus durionis* and *Fructobacillus ficulneus* (*p* < 0.05, ANOVA, Mann–Whitney, Bonferroni corrected). At the same time, the relative abundance of wine spoilage bacteria *Oenococcus kitaharae* and *Oenococcus oeni* significantly reduced at 7–10 days of tapping. Similarly, a significant gradual increase in the relative abundance of *Moraxellaceae* reflected with a rise in *Acinetobacter xiamenensis* during ron palm wine tapping days. In addition, we noticed a considerable decline in the relative abundance of the members of *Acetobacteriaceae*, particularly *Acetobacter tropicalis*, *Gluconobacter frateurii*, and *Gluconobacter oxydans,* during ron palm wine tapping (*p* < 0.05, ANOVA, Mann–Whitney, Bonferroni corrected). Contrary to ron palm wine, a decline in the relative abundance of the members of *Leuconostocaceae*, particularly *Fructobacillus durionis*, *Fructobacillus ficulneus* and *Fructobacillus fructosus* was noticed up to 10–15 days of oil palm wine tapping (Figure 3B). In addition, the relative abundance of *Lactobacillus*, particularly *Lactobacillus diolivorans* and *Lactobacillus fermentum,* also declined during the later stages of oil palm wine tapping. At the final stage of the tapping process (30 days), the relative abundance of *Acetobacter* became the dominant (58.33%), surpassing the *Lactobacillus*, whereas in ron palm wine tapping *Lactobacillus* maintained as the dominant (80% relative abundance) up to the final stage of tapping (25 days).

### Dynamics in yeast communities during palm wine tapping

**Figure 4:**
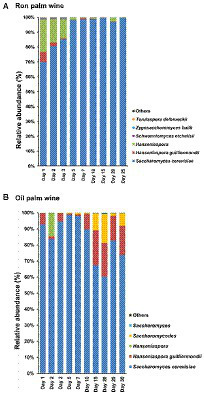
The yeast taxon bar chart shows the dynamics in the relative abundance (%) of predominant yeast species during ron palm wine **(A)** and oil palm wine **(B)** tapping days. The taxa with a mean relative abundance of <1% across the samples are combined and shown as others.

The predominant yeast taxa associated with the tapping of both ron palm wine and oil palm wine fermentation were *Saccharomyces cerevisiae* (with an average relative abundance of 91.8% and 83.5%, respectively) and *Hanseniaspora* (with an average relative abundance of 7.6% and 10.5%, respectively). However, the yeast dynamics analysis showed an opposite trend in ron palm wine and oil palm wine tapping (Figure 4). Ron palm wine samples showed a high relative abundance of *Hanseniaspora* sp. during the early stage of tapping (with an average relative abundance of 16.8%, 1–3 days), which further declined in the later stages of tapping (with an average relative abundance of 0.8%, 5–25 days; *p* = 0.0085, ANOVA, Mann–Whitney, Bonferroni corrected). Overall, we observed a complete dominance of *Saccharomyces cerevisiae* (with a relative abundance of 98.7%) during the later stages (5–25 days) of ron palm wine tapping (*p* = 0.0027). On the contrary, the relative abundance of *Hanseniaspora guillermondii* was high during later stages of oil palm wine tapping (with an average relative abundance of 17%, 10–30 days) in comparison to early stages (with an average relative abundance of 3%, 0–7 days; *p* = 0.0164). The unique presence of *Saccharomycodes* sp. in oil palm wine fermentation increased from 0.2% of relative abundance during early stages (0–7 days) to 7.5% of relative abundance in the later stages (10–30 days; *p* = 0.0319, ANOVA, Mann–Whitney, Bonferroni corrected). The dominance of *Saccharomyces cerevisiae* in early stages of fermentation in oil palm wine tapping and in later stages of fermentation in ron wine tapping, vice versa for *Hanseniaspora* predominance, was the key feature differentiating the yeast dynamics in ron palm wine and oil palm wine tapping in Côte d’Ivoire.

### Metabolite changes during palm wine tapping

**Figure 5:**
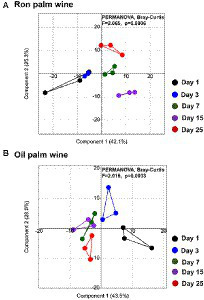
PCoA using Bray–Curtis dissimilarity based on the palm wine metabolites profile generated by LC-HRMS shows dynamics during ron palm wine **(A)** and oil palm wine **(B)** tapping days (1, 3, 7, 15 and 25 days). The significance in the difference is expressed as Bonferroni corrected *p*-values (*p* = 0.0006, *F* = 2.065, PERMANOVA).

We analyzed the changes in the metabolites during the palm wine tapping days by liquid chromatography-high resolution mass spectrometry (LC-HRMS). The quality-filtered LC-HRMS metabolites data assigned to the identity of the possible compounds by the HMDB/KEGG database resulted in a total of 338 metabolites. Both palm wines’ metabolite composition enriched with organic acids, hexose deoxy sugars, sugar alcohols and sugar acids, several amino acids and terpenes. PCoA using Bray–Curtis dissimilarity analysis based on the overall metabolite profile showed a minor change in the produced metabolites in ron palm wine and oil palm wine over 25 days of tapping (*p* < 0.005, *F* ~ 2.0, PERMANOVA; Figure 5). Identity of the significantly differing metabolites over different stages of ron palm and oil palm wines tapping are shown in Table 1 and their changes in Supplementary Figure 1. We observed a significant decline in the relative abundance of essential amino acids, DL-Methionine present in ron palm wine and DL-Tryptophan present in oil palm wine during the later stage of tapping (15–25 days). We noticed a drastic reduction in gevotroline (−4.83, log 2 fold relative abundance change) during later days in ron palm wine tapping. In addition, the metabolite named 2,2,2-Trifluoroethanol, a potential toxin, reduced drastically (−5.18, log 2 fold relative abundance change) in the later stage (25 days) of ron palm wine tapping. However, a metabolite with the closest identity to Sesartemin significantly increased (6.04, log 2 fold relative abundance change) at 15 days of ron palm wine tapping. Changes in metabolite profile were minimal in the two palm wine tapping studied, but more changes were visible in ron palm wine tapping.

**Table 1:** Identities of the significantly differing metabolites over different stages of ron palm wine and oil palm wine tapping, by LC-HRMS analysis.

Types of palm wine	ID	RT (min)	MW	MZ	Changes (log 2 fold change of relative abundance in comparison to the first day of tapping)	Closest Chemspider hits (within 20 ppm)
Ron palm wine	C43	5.95	100.01	5.95	−5.18, reduced in the last stage (25 days) of tapping	2,2,2-Trifluoroethanol, Succinic anhydride
	C166	13.42	149.05	148.04	−1.45, reduced in the last stage (15–25 days) of tapping	DL-Methionine
	C333	4.34	220.15	219.14	1.46, increased in the later stage (15–25 days) of tapping	3,5-di-tert-butylbenzoquinone; a,a-Dimethylphenethyl butyrate; 2-Phenylethyl caproate; 3-Phenylpropyl isopentanoate; 2,2,5,7,8-Pentamethyl-6-chromanol; (3aS,5aR,7S,9aS,9bR)-7,9b-Dimethyl-3a,5a,6,7,8,9,9a,9b-octahydronaphtho[1,2-c]furan-1(3H)-one; Hexyl phenylacetate;(3R,4S,4aS,6R)-3-Hydroxy-6-isopropenyl-4-methyl-4,4a,5,6,7,8-hexahydro-2(3H)-naphthalenone;13-Tetradecene-1,3-diyne-6,7-diol; 3-Acetyl-4a,5-dimethyl-4a,5,6,7,8,8a-hexahydro-2(1H)-naphthalenone; (6Z)-6-Tetradecene-1,3-diyne-5,8-diol
	C563	12.17	345.14	344.14	−4.83, reduced in the later stage (15–25 days) of tapping	Gevotroline hydrochloride
	C693	10.07	430.17	10.07	6.04, increased at 15 days of tapping	Sesartemin; (1R,2S,4S,10R,12R,14R,15R)-7-Formyl-4-isopropenyl-12,14-dimethyl-17-oxo-11,16,18,19-tetraoxapentacyclo[12.2.2.1~6,9~0.0~1,15~0.0~10,12~]nonadeca-6,8-dien-2-yl acetate;4-Methoxy-6-[4-(3,4,5-trimethoxyphenyl)tetrahydro-1H,3H-furo[3,4-c]furan-1-yl]-1,3-benzodioxole; 3-[(7-Methoxy-1,3-benzodioxol-5-yl)methyl]-4-(3,4,5-trimethoxybenzyl)dihydro-2(3H)-furanone;1-(3,4-Dimethoxyphenyl)-4-(4-hydroxy-3-methoxyphenyl)dihydro-1H,3H-furo[3,4-c]furan-3a(4H)-yl acetate
	C734	17.34	463.19	462.18	−0.57, reduced in the later stage (15–25 days) of tapping	(5alpha,6beta)-3-Hydroxy-17-methyl-4,5-epoxymorphinan-6-yl beta-L-glucopyranosiduronic acid;1-O-[(1,8-Diethyl-1,3,4,9-tetrahydropyrano[3,4-b]indol-1-yl)acetyl]-D-glucopyranuronic acid
Oil palm wine	C289	13.34	204.09	203.08	−0.52, reduced in the later stage (15–25 days) of tapping	DL-Tryptophan
	C356	2.83	227.98	226.97	1.01, increased in the later stage (15–25 days) of tapping	Ammonium persulfate

## Discussion

The microbial diversity and dynamics during palm sap tapping days are of considerable interest in understanding the microbiological processes, characteristics of palm wine and controlling palm wine quality. The palm sap fermentation process is controlled by a complex microbial community that evolved during natural fermentation. Few studies using next-generation sequencing techniques showed dynamics in yeast and bacterial communities during laboratory fermentation of palm sap for 36–96 h (Astudillo-Melgar et al., 2019; Das and Tamang, 2021). Whereas microbial dynamics during palm sap tapping in the field over 25–30 days is still not established through the view of next-generation sequencing techniques. The stage-wise palm wine samples collected during tapping days contained 504 bacteria and 95 yeast species in the present study. Similar to earlier studies, yeast belongs to *Saccharomyces cerevisiae* and *Hanseniaspora* spp.; and bacteria belong to *Lactobacillus*, *Leuconostoc*, *Fructobacillus*, *Acetobacter*, and *Glucanoacetobacter* were the leading players during the tapping of two palm wines studied here (Amoa-Awua et al., 2007; Astudillo-Melgar et al., 2019; Djeni et al., 2020; Das and Tamang, 2021). However, we noticed an opposite tendency of their occurrence and dynamics; the changes reflected a decline in pH with low microbial diversity during tapping days in oil palm wine; whereas increased pH with high microbial diversity in ron palm wine tapping. The distinct changes in microbial diversity and pH observed during ron and oil palm wine fermentation over 25–30 days support the microbial community dynamics during palm wine tapping.

The yeast community associated with both types of palm wine tapping can be arbitrarily divided into *Saccharomyces* and non-*Saccharomyces* groups. In agreement with previous studies, *S. cerevisiae* dominates the alcoholic fermentation of palm sap (Brown, 1994; Amoa-Awua et al., 2007). The dominance of *Saccharomyces cerevisiae* in early stages of fermentation and non-*Saccharomyces* (*Hanseniaspora* spp.) in the later stages of oil palm wine tapping, vice versa in ron palm wine tapping, and unique presence of *Sacchomycodes* in the later stages (15–30 days) of oil palm wine tapping were the key differences in yeast dynamics observed during palm wine tapping. Usually, non-*Saccharomyces* yeasts were present substantially during the early stages of wine fermentation, and the majority disappeared during the later stages due to vigorous alcoholic fermentation by *Saccharomyces cerevisiae*, similar to ron wine tapping (Fleet et al., 1984; Jackson, 1994; Henick-Kling et al., 1998). Meanwhile, recent works in grape wine fermentation have shown that some non-*Saccharomyces* yeasts can survive to the later stages of fermentation than initially believed (Longo et al., 1991; Granchi et al., 1998; Fleet, 2003; Combina et al., 2005), a similar phenomenon observed here in the oil palm wine tapping. Similar to our results, domination of *Saccharomyces* and *Hanseniaspora* during palm wine fermentation (Amoikon et al., 2020; Das and Tamang, 2021) and *Saccharomycodes* in oil palm wine tapping (Stringini et al., 2009) were reported in the earlier studies. The indigenous non-*Saccharomyces* yeasts present during the first few days of palm wine fermentation may contribute to the palm wines’ desirable aromatic properties (Lema et al., 1996). Production of a higher amount of volatile organic compounds by *Hanseniaspora guilliermondii* and *Hanseniaspora jakobsenii*, the dominant yeast isolated from raffia palm wine and ron palm wine, was reported as the reason for the unique palm wine flavor (Gamero et al., 2016; Lombardi et al., 2018; Amoikon et al., 2020). Moreover, the sequential culture inoculation of *Hanseniaspora guilliermondii* and *Saccharomyces cerevisiae* gave a better aroma and quality in Campanino white grape wine (Lombardi et al., 2018). In addition, the metabolic products resulting from non-*Saccharomyces* yeast like higher alcohols (Heard, 1999; Zohre and Erten, 2002; Clemente-Jimenez et al., 2004) can affect the overall quality of the palm wine.

In bacteria, fructophilic lactic acid bacteria (FLAB), mainly *Fructobacillus* and *Leuconostoc*, encountered during both types of palm wine tapping is due to their affinity to the fructose enriched habitat such as flowers, palm sap and honey bee gut (Endo et al., 2018). FLAB was reported as the primary bacteria of tuba fermentation (palm wine from coconut tree) in Mexico (Astudillo-Melgar et al., 2019). However, *Fructobacillus* has not been detected in the toddy produced from *Phoenix sylvestris* in India (Das and Tamang, 2021). As honey bees are the primary source of *Fructobacillus*, the absence of *Fructobacillus* in the Indian palm wine indicate the change in the insect ecology in the study area. Moreover, FLAB is more sensitive to acid stress (McDonald et al., 1990; Van der Meulen et al., 2007), which can explain the difference in their relative abundance during later stages of ron palm and oil palm wine tapping observed in this study. A decline of FLAB at later stages of oil palm wine tapping, whereas an increase in the later stages of ron palm wine tapping, was noticed in our study. Moreover, the decrease or change of fructose during the later stages of 22 days of raffia palm wine tapping reported in an earlier study (Faparusi, 1981) may be one more reason for FLAB dynamics. Oil palm wine was reported to develop a vinegary taste during later stages of tapping (Stringini et al., 2009), which may be due to the predominance of acetic acid bacteria (*Gluconobacter* and *Acetobacter*) surpassing *Lactobacillus* during the last days of tapping (30 days; Amoa-Awua et al., 2007; Santiago-Urbina and Ruiz-Teran, 2014). Moreover, the palm wine from both types of tapping contained a substantial portion of uncultured bacteria during the early days of tapping which is consistent with results of Djeni et al. (2020); but they continuously decreased until the end of the tapping process in both types of palm wine.

Many factors like palm tree species which influence the sap composition, geographic location, and climatic conditions could be the basis of the difference in the microbial community dynamics observed during the two types of palm wine tapping in Côte d’Ivoire. However, as Tapsoba et al. (2014) suggested, the most relevant reason for this difference is the indigenous tapping process practiced. Indeed, in Côte d’Ivoire, generally for ron palm tapping, the sap is obtained from a live standing tree at the height of more than 10 m, thus limiting the sources of microbes which are mainly insects and the materials used for tapping; that is not the case for oil palm wine tapping, where the tree is felled or killed before and the tapping process is achieved in a more contaminated environment. According to previous studies, the main component of palm sap is sugar (about 15%; Amoa-Awua et al., 2007; Sudha et al., 2019; Oladoja et al., 2021). A depletion in this sugar content in oil palm sap may occur because of the fact that the trees are felled, and the leaves are cut off; hence the palm no longer performs photosynthesis and does not produce anymore sugars (Santiago-Urbina et al., 2013). Similarly, Faparusi (1981) reported a decline in sucrose, glucose, and fructose levels during raffia palm wine tapping. On the other hand, in oil palm wine, the total sugars in the samples dropped from initial concentrations of about 14%–8% during the 35 days of tapping period (Amoa-Awua et al., 2007). Therefore the sap composition differs in the later stages of tapping, leading to the domination of non-*Saccharomyces* yeasts and acetic acid bacteria in the later stages of oil palm wine tapping.

The primary biochemical characteristics of palm wine are resultant from natural sap, and analyzing the chemical composition of palm wine help to assess the nutritional values and health benefits of palm wines. Our earlier study observed a clear difference in the metabolite profile of three palm wines types produced in Côte d’Ivoire (Djeni et al., 2020). In comparison to microbial community dynamics over palm wine tapping, the changes in the metabolite composition during palm wine tapping days are very much limited in both types of palm wines during this study. Despite the fact that our analysis by LC-HRMS detected hundreds of components, it was difficult to fix the identity of several metabolites due to the limitations in available spectra and possible novel compounds. A recent study on raffia palm wine using LC–MS also identified several compounds, mainly polyphenols and their glycosides, vitamins, and amino acids (Erukainure et al., 2019). These metabolites undoubtedly affect the wine quality and contribute to set up the characteristics of the palm wine produced during tapping days. This metabolite profiling is indispensable to consumers and food producers interested in claiming palm wine as a functional or medicinal beverage. Among the identified compounds, several essential amino acids are abundantly present in palm wine (Ibegbulem et al., 2013). The Methionine not detected in the coconut palm sap called “Neera” (Sudha et al., 2019) was found in the palm wine. However, our study observed a reduction of DL-Methionine present in ron palm wine and DL-Tryptophan present in oil palm wine during the later tapping stages (15–25 days). Tryptophan, the least abundant in everyday food and precursor for serotonin, can support palm wine as a hypnotic and an antidepressant. The higher relative concentration of gevotroline (an antipsychotic agent and a dopamine receptor D2 antagonist) present in the early stages of oil palm wine tapping and a drastic decline during later stages of tapping support the difference in the health benefits of the palm wine obtained from different stages of tapping. This indicates early stages of tapping are more nutritional and healthy than the later stages.

## Conclusion

In this study, next-generation sequencing-based analysis of stage-wise collected samples clearly showed dynamics in bacterial and yeast communities during ron palm wine and oil palm wine tapping over 25–30 days. However, we viewed an opposite trend of microbial dynamics while comparing ron palm wine and oil palm wine. This microbial dynamics may be a potential indicator of changes in metabolic activities during palm sap fermentation throughout the tapping process, thus contributing to establish particular features of palm wines in different stages of tapping. This understanding of changes in the microbial ecology and metabolites profile during palm wine tapping could help improve the industrial production of palm wine and ensure the quality and safety, and also useful to identify biomarkers to assess the quality of the beverage and help design an optimum starter culture consortium.

## Data availability statement

The datasets presented in this study can be found in online repositories. The names of the repository/repositories and accession number(s) can be found at: https://www.ncbi.nlm.nih.gov/, PRJNA507709.

## Author contributions

TD, MD, and KJ designed the study. KK, FA, and LA collected samples. TD did all pre-sequencing works for the Illumina. SK, NT, and RL processed the data. KJ analyzed the data. CA, TD, and KJ wrote the manuscript. All authors contributed to the article and approved the submitted version.

## Funding

This work was financially supported by funds from DST-CV Raman fellowship agency (INT/NAI/CVRF/2014), the Institute of Bioresources and Sustainable Development (IBSD) and the International Foundation for Sciences (E/5669-l).

## Conflict of interest

The authors declare that the research was conducted in the absence of any commercial or financial relationships that could be construed as a potential conflict of interest.

## Publisher’s note

All claims expressed in this article are solely those of the authors and do not necessarily represent those of their affiliated organizations, or those of the publisher, the editors and the reviewers. Any product that may be evaluated in this article, or claim that may be made by its manufacturer, is not guaranteed or endorsed by the publisher.
