# Supplementary material for: Dynamics of microbial populations and metabolites of fermenting saps throughout tapping process of ron and oil palm trees in Côte d’Ivoire
Source: Front Microbiol. 2022 Oct 31;13:954917. doi: 10.3389/fmicb.2022.954917 (PMC9660251; doi:10.3389/fmicb.2022.954917)
Supplement: Supplementary file 1 [file Table_1.DOCX]

**Supplementary figure**

**A**

**B**

**Supplementary Figure 1**. Relatives abundances of the metabolites significantly differing throughout the tapping process (Day 0 to Day 25) of *Borassus aethiopum* (A) and *Elaeis guineensis* sap (B).
